# Self-reported prevalence of hand eczema and associated factors among hair dressers of Debre Berhan City in North Eastern Ethiopia

**DOI:** 10.1371/journal.pone.0336974

**Published:** 2025-11-25

**Authors:** Belachew Tekleyohannes Wogayehu

**Affiliations:** Department of Environmental Health, Debre Berhan Health Science College, Debre Berhan, Ethiopia; Kampala International University - Western Campus, UGANDA

## Abstract

**Introduction:**

The prevalent condition known as hand eczema has been associated with substantial decreased quality of life, as well as considerable social and occupational expenses. Even though hairdressing is a significant source of wealth, it is linked to several kinds of medical problems mainly skin conditions. Limited studies conducted in Ethiopia to assess self-reported prevalence of hand eczema and associated factors.

**Objective:**

This study aims to assess self-reported prevalence of hand eczema and associated factors among hairdressers of Debre Berhan city.

**Methods:**

A cross-sectional study was conducted among 435 hairdressers of Debre Berhan city in North Eastern Ethiopia from January 10 to February 20, 2025. A simple random sampling technique was used to select hair dressers. Data was collected using a structured questionnaire adapted from Nordic occupational skin questionnaire and observational checklist through face to face interview and observation. Multivariable binary logistic regression was employed to identify associated factors of hand eczema.

**Results:**

Prevalence of hand eczema among hairdressers of Debre Berhan city was 56.9%. Poor knowledge (AOR = 2.89, 95% CI: 1.199–4.963), not utilizing personal protective equipment consistently over the years (AOR = 3.8, 95% CI: 2.183–7.012), low hand washing frequency per day (AOR = 3.4, 95% CI: 1.399–6.433) and not taking OHS training (AOR = 4.8, 95% CI: 2.617–8.709) were identified factors of hand eczema.

**Conclusions:**

Prevalence of hand eczema among hair dressers in Debre Berhan city was high. Poor knowledge, not utilizing personal protective equipment consistently over the years, low hand washing frequency per day and not taking OHS training were identified factors. Hair dressers should utilize proper type of personal protective equipment before starting any activities in the work place. Inclusion of hand eczema education in Technical and Vocational Educational and Training (TVET) or policy-level interventions would enhance occupational health awareness, early prevention strategies and long-term skin protection practices among hairdressers.

## Introduction

According to international statistics, occupational skin diseases rank second in frequency among occupational diseases, after musculoskeletal disorders [1]. Hand eczema (HE) or hand dermatitis is a skin condition that is exclusive to the hands and causes inflammation [2]. Workers such as caterers, dry cleaners, metalworkers, auto mechanics, nurses, cleaners, painters, and hairdressers who use products like paints, soaps, detergents, shampoos, hair relaxants, colors, industrial chemicals and solvents on a daily basis at work are among the occupants with the highest rates of hand eczema [3]. Dermatology facilities frequently see patients with persistent hand eczema, many of whom have severe cases that don’t respond to standard therapy [4]. Hairdressing include, but is not limited to, the cutting, cleaning, dyeing, style, and arranging of hair. These hairstyling services are provided to improve the appearance, self-esteem and self-image of the consumer [5]. Several million hairdressers face various occupational health risks. These workers could not be following the safety procedures necessary to prevent and control any potentially harmful effects of the thousands of hazardous compounds present in these cosmetics, particularly in developing countries [6]. Approximately 60% of patients with atopic dermatitis have involvement of the hands adding to the burden of disease [7]. Nowadays, many people color their hair, which exposes them to powerful contact allergens like p-phenylenediamine and professionals in the hairdressing industry often come into dangerous chemicals and skin irritants [8]. Regular exposure to these chemicals can cause respiratory, reproductive and skin disorders as well as other work-related diseases [4]. Different studies conducted in United States of America (USA), United Kingdom (UK), Croatia and Nigeria showed 70% [9], 72.7% [10], 50.3% [11] and 34.5% [6] prevalence of hand eczema. Hairdressers’ career length is shortened by Occupational Hand Eczema (OHE), especially if they relapse frequently. OHE increases a hairdresser’s risk of leaving the industry by 20% and reporting OHE ‘almost all the time’ increases that risk by 90% which means that the career length will be shortened by two and seven years respectively [11].

According to the findings of different studies, Being woman, younger age and job experience were strongly associated with hand eczema among hairdressers [12]. Despite hairdressing being an important source of income and wealth, hairdressing is associated with various health problems such as high social and occupational costs as well as a significantly reduced quality of life [13]. According to earlier research, due to a lack of awareness and attitudes, hairdressers fails to conform to the proper occupational health and safety protocols [5]. Even though working conditions and safety measures are still in their infancy, research conducted in Addis Ababa, Ethiopia, revealed that employees at small and medium-sized businesses including beauty salon are overlooked in health and safety programs [14]. However, in earlier research on hairdressers in Ethiopia, scientists frequently focused exclusively on chemical and biological hazard exposures. There are no literatures in prevalence of hand eczema including assessing knowledge, attitude and practice of respondents towards safety practice. Little to no health supervision in Ethiopia, or healthcare facilities not pay attention and also practically neglect hairdressers. Furthermore, there is a lack of information regarding the prevalence and prevention practice of hand eczema among hairdressers. Investigation of the extent and causes of the disease and knowing prevalence plays a critical role in determining effective prevention and control strategies. Finally, the findings of the current study assist in the achievement of Sustainable Development Goal 8 (SDG 8), which says “promote inclusive and sustainable economic growth, employment and decent work for all”. Therefore, this study aimed to determine the prevalence of hand eczema among hair dressers and associated factors of Debre Berhan city in North Eastern Ethiopia.

## Methods

### Study setting

Debre Berhan is a city in the central Ethiopian highlands at an altitude of 2,840 m in North Shewa Zone of Amhara Region, about 130 km northeast of Addis Ababa. The city had a total population of 160,408 living in five sub-cities in 2020 G.C; 73,929 were males and 86,479 females. The average annual temperatures during the day and night are 20.7°C and 8.2°C. Average annual rainfall is 1219 mm and the mean relative humidity is 31% [15].

### Study design and period

A cross-sectional study was employed to assess self-reported prevalence of hand eczema and associated factors among hairdressers of Debre Berhan city in North Eastern Ethiopia from January 10 to February 20, 2025.

### Source population, inclusion and exclusion criteria

All hairdressers working in beauty salon in Debre Berhan city constituted the source population and all hair dressers in each sub-city made up the study population. Hair dressers who had worked in beauty salon with at least one year experience were included in the study. Hair dressers working in beauty salon who had any allergy, on medication due to confirmed hand eczema, history of smoking, history of alcohol intake and personal/family history of hand dermatitis were excluded from the study.

### Sample size determination and sampling procedure

The sample size for this study was computed by using single population proportion formula by considering the prevalence of hand eczema among hair dressers as 21.5% [16,17].


$$n=\frac{({Z\alpha /2)}^{2}\times P\left(1-P\right)}{d^{2}}$$

Where, n = is the required sample size

   Z = 95% confidence interval (Zα/2 value =1.96)

   P = Prevalence of hand eczema (21.5%)

   d = 5% margin of error


$$n=\frac{\left({\text{1}.\text{9}\text{6})}^{\text{2}}\times \text{0}.\text{2}\text{1}\text{5}(\text{1}-\text{0}.\text{2}\text{1}\text{5}\right)}{({\text{0}.\text{0}\text{5})}^{\text{2}}}=\frac{\text{3}.\text{8}\text{4}\times \text{0}.\text{2}\text{5}}{\text{0}.\text{0}\text{0}\text{2}\text{5}}=\;259$$

Finally, by considering 10% of non-response rate, the final sample size for this study was 259 + 38.4 = 285.

Sample size for an associated factor was calculated by using Epi Info Version 7.2 software by considering age of study participant which is significant factor and consider power 80%, Ratio 1:1 and Adjusted Odds ratio 1.76 and the final sample size by considering 10% non-response rate was 435 which is the sample size of the current study.

**Fig 1:**
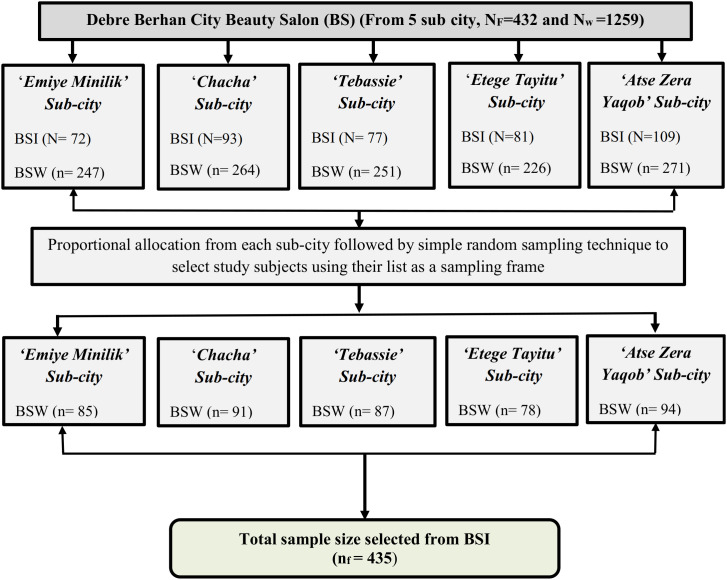
Schematic presentation of sampling procedures to select study participants in Debre Berhan city, 2025 (NF: Total number of beauty salon institutions in the city, Nw: Total number of beauty salon workers in the city, BSI: Beauty Salon Institutions, BSW: Beauty Salon Workers).

Total number of beauty salon institutions and hair dressers in each sub-city were obtained from each sub-city’s administration, office of Micro, Small and Medium Enterprises (MSMEs). Overall, there are 1259 hairdressers in Debre Berhan city beauty salon hair dressers and there are about 432 hair dressing institutions in the city. Then after, proportional allocation was conducted to get the required number of hair dressers from each sub-city for the study. List of all hair dressers in each sub-city was obtained from each sub-city administration, office of MSMEs for selection of study subjects. Since the institutions were small-scale, workers did work all types of work type so that there is no regular division of work by work type. Finally, using list of hair dressers from each sub-city as a sampling frame, required number of hair dressers were selected by using simple random sampling technique (Fig 1).

### Outcome and explanatory variables

**Outcome variable**

- Hand eczema (Yes/No)

**Explanatory variables**

- Socio-Demographic Factors: Age, marital status, educational status and family size
- Behavioural Factors: Knowledge, attitude, practice, utilization of personal protective equipment and hand washing frequency
- Occupational Factors: Work experience, number of working days per week and Occupational health and Safety training

### Operational definitions

#### Hand eczema.

The development of one or more of the symptom/s of redness, dry skin with scaling/flaking, fissures or cracks, weeping or crusts, tiny water blisters (vesicles), papules, rapidly appearing itchy wheals/welts, itching, burning, prickling, or stinging, tenderness and aching/pain which lasts at least one or three months or recurs at least twice a year [18].

#### Hairdressers.

Hairdressers are defined as individuals who are actively engaged in hairdressing activities as their primary occupation in Debre Berhan city. This includes individuals involved in cutting, styling, coloring, and treating hair in salons or other hairdressing establishments.

#### Prevalence.

Prevalence is defined as the percentage of hairdressers in Debre Berhan city who have hand eczema during data collection.

#### Good knowledge.

In this study, respondents were asked 11 questions, and those who scored greater than or equal to the mean value were considered to have “good knowledge” and those who scored less than the mean value were considered to have “poor knowledge”.

#### Positive attitude.

In this study, respondents were asked 9 questions, and those who scored greater than or equal to the mean were categorized as having a “positive attitude,” while those who answered less than the mean were considered to have a “negative attitude”.

#### Good practice.

In this study, respondents were asked 10 questions, and those who scored greater than or equal to the mean were categorized as having “good practice” while those who answered less than the mean were considered to have “poor practice “.

### Data collection tools and procedures

Data was collected with the use structured questionnaire adapted from Nordic occupational skin questionnaire with some modification [18]. Data was collected through face to face interview and direct observation of work place characteristics/work area. The questionnaire (annex) consists of six parts. Part I: consists of questions about socio-demographic and economic characteristics of hair dressers, Part II: consists of questions about knowledge assessment questions of hair dressers, Part III: consists of questions about attitude assessment questions of hair dressers, Part IV: consists of questions practice assessment questions of hair dressers, Part V: consists of questions about occupational characteristics of hair dressers and consists of observational checklist to assess the working conditions of beauty salon. Four data collectors with profession of two BSc degree in Environmental Health and two BSc Nurse and one supervisors (senior Environmental Health) were participated in this study. Observational checklist was also used to assess the characteristics of work place.

### Data quality management

To ensure data quality, a structured questionnaire adapted from Nordic occupational skin questionnaire [18] was used. The questionnaire was first translated from English to Amharic by two independent language experts (in to local language commonly spoken by communities) and then back again to English by a third translator to ensure its consistency and semantic equivalence with the original data collection tool. Following language translation, the questionnaire was reviewed by senior occupational health experts and environmental health professionals to assess its content validity and contextual appropriateness to the local population. Then after, pre-test was conducted among 10% of the total sample size in Shewarobit town with almost similar demographic and occupational characteristics through random selection of hair dressers. Through this procedure, it was made possible to make sure the modified instrument was appropriate for the local population and could gather useful information about occupational exposures and hand eczema in the population of interest. All data collectors and supervisors got two days of training by the principal investigator and performed practical exercises to be familiar with the data collection tool. One senior health professionals (Environmental and Occupational Health) was assigned in this study as a supervisor for data collection to monitor the data collection process regularly on questionnaire. The questionnaire was verified on a regular basis for completeness and consistency by each data collector. Incomplete data due to data collector’s problems was recollected again and incomplete data due to withdrawal of study participants from the interview, and unwillingness of study participants for the study was considered as non-response. Finally, all the collected data was become cleaned and cross-checking was made before analysis.

### Data management and data analysis

At the end of each day of data collection, the data was verified for completeness and missing values. Data obtained from the interview on hand eczema was entered in to Epi-Data version 4.6 before being exported into STATA version 14 for descriptive and factor analysis. Then after, data was exported in to STATA version 14 software. Once data was exported, it was checked for any missing values. Moreover, basic quality assurance measures was performed using descriptive statistics results from cross-tabulations (contingency coefficient) and frequency distributions before doing statistical analysis. After that, categorization and re-categorization of variables was conducted for both continuous and categorical independent variables. Finally after ensuring the quality of data, descriptive statistics was employed to summarize socio-demographic characteristics of the study participants using frequency distribution, measures of central tendency and dispersion that was displayed using tables and figures depending on the normality of data (i.e., if the data was normally distributed, mean and standard deviation as a measure of central tendency and dispersion was used respectively and if not, median and interquartile range was used).

The Homer and Lemeshow test was used to determine the model’s overall goodness. P value of greater than 0.05 (P > 0.05) implies a strong fit to the data and overall model fitness. In this study, Hosmer and Lemeshow test come up with P value of 0.328 (P; 0.328 > 0.05) which is insignificant. Since the null hypothesis was accepted, logistic assumption was not violated which indicates good model fitness. Again Nagelkerke R Square was also employed in this study to determine how much percent of the variability of dependent variable was explained by independent variables used for the study. The value of Nagelkerke R Square in this study was 0.652. This means 65.2% of the variability of hand eczema among hair dressers was explained by the variables entered in this study. To test multicollinearity between independent variables, both Variance Inflation Factor (VIF) and Standard Error (SE) was utilized. All variables in this study had VIF < 5 and −2 < SE < 2 which indicates no problem of multicollinearity between independent variables. Analysis of data with respect to factors affecting hand eczema was conducted depending on the nature of variables. Bivariable binary logistic regression model was employed to determine the Crude Odds Ratio (COR) and 95% CI for the association between dependent and independent variables. After that, those variables that had P < 0.25 in the Bivariable binary logistic regression were candidate variables to be entered into multivariable binary logistic regression to adjust the possible effect of confounders. Based on the result of Adjusted Odds Ratio (AOR) at 95% CI, variables having P < 0.05 was considered as statistically significant predictors of hand eczema.

### Ethics approval and consent to participate

Ethical clearance was taken from Ethical Review Committee of Debre Berhan Health Science College (Ref no. DBHSC_1426/2017). An official letter was written to Debre Berhan City department of Labor and Social Affairs and offices of MSMEs in each sub city. Permission letter was also obtained from office of MSMEs requesting facilitation to conduct a research (Ref no. ኢ/ኤ/አ/ሥ/ቡ/113/2017). The aim and method of the study and importance of their participation was clearly stated to each study participants. Participants in the study who fulfill the criteria for the study and agreed to participate was given an informed Amharic written consent and signed before data collection. The respondents were informed that they have full right to withdraw at any time when they feel discomfort. Confidentiality of information given by each respondent was properly maintained by avoiding possible identifiers such as name of the study participants. Instead, identification number was used as a reference and anonymity was explained clearly for the participant. Participants of the study had not get a direct benefit such as money but they become a beneficiary in the future from the study, as the study will help concerned bodies to prepare intervention measures for prevention of hair dressers health. During data collection, those who showed sign of chronic hand eczema were linked to health facilities found in the city to seek more clinical diagnosis and treatment. All the above written consent is based on the Helsinki declaration of ethical principles for medical research involving human subjects [19].

## Results

Out of 435 hair dressers selected for this study, 406 hair dressers were participated in this study with a response rate of 93.3% by hair dressers.

### Socio-demographic characteristics of hair dressers

Out of 406 hair dressers participated in this study, majority 217 (53.5%) of hair dressers were age of between 25–39 years. Moreover, out of 406 hair dressers, majority 213 (52.5%) of hair dressers were single (Table 1).

**Table 1:** Socio-demographic characteristics among hair dressers of Debre Berhan city, January 10-February 20, 2025.

*Variables*	*At least one hand eczema symptom*		*Frequency*	*Percentage*
	Yes	No		
Age (years)				
≤ 24 years	49	47	96	23.6%
25-39 years	118	99	217	53.5%
≥ 30 years	54	39	93	22.9%
Sex				
Male	56	165	221	54.4%
Female	66	119	185	45.6%
Marital Status				
Single	121	92	213	52.5%
Married	95	39	134	33%
Divorced	22	25	47	11.5%
Widowed	6	6	12	3%
Educational Status				
Able to read and write	55	32	87	21.4%
Primary education	93	56	149	36.7%
Secondary education	58	48	106	26.1%
Diploma and above	28	36	64	15.8%
Family Size (number)				
≤ 2	137	106	243	59.8%
3-4	61	42	103	25.4%
≥ 5	36	24	60	14.8%

### Knowledge, attitude and practice of hair dressers

#### Knowledge of hair dressers.

Out of 406 hair dressers participated in this study, 122 (30.1%) had good knowledge towards hand eczema whereas the remaining 284 (69.9%) had poor knowledge towards hand eczema (Table 2).

**Table 2:** Knowledge towards hand eczema among hair dressers of Debre Berhan city, January 10-February 20, 2025.

S/N	Questions	Category	Frequency (n)	Percentage (%)
1	What is hand eczema?	Causing the skin on the hands to be dry and cracked	110	27.1%
		Skin irritation causing redness, itching, and peeling	87	21.4%
		A fungal infection affecting the hands	127	31.3%
		A viral rash on the hands	82	20.2%
2	Which of the following is a common cause or trigger for hand eczema?	Exposure to chemicals used in hairdressing	91	22.4%
		Lack of hand-washing	147	36.2%
		Exposure to heat only	72	17.7%
		Infection by bacteria	96	23.7%
3	In which of the following professions is hand eczema most common?	Office workers	77	19%
		Hairdressers and cleaners due to frequent exposure to water, soaps, and chemicals	79	19.5%
		Teachers	96	23.6%
		Drivers	154	37.9%
4	What are common symptoms of hand eczema?	Itching, redness, dryness, and sometimes blisters or cracks	103	25.4%
		Pain and swelling only	99	24.4%
		Fever and rash	87	21.4%
		No symptoms, only pain	117	28.8%
5	Which of the following environmental factors increases the risk of hand eczema among hairdressers?	Dry and cold climate	154	37.9%
		Exposure to humidity and harsh chemicals in hair dyes and treatments	66	16.3%
		High altitude	83	20.4%
		Only exposure to sunlight	103	25.4%
6	Which of the following is a significant risk factor for hand eczema in hairdressers?	Wearing gloves for protection	50	12.3%
		Prolonged exposure to water and irritants like hair dyes	259	63.8%
		Working in air-conditioned environments	41	10.1%
		Not using hair products	56	13.8%
7	How does constant exposure to water and chemicals affect a hairdresser’s skin?	It can cause skin dryness and irritation, which may lead to hand eczema	145	35.7%
		It has no impact on the skin	102	25.2%
		It helps keep the skin moisturized	70	17.2%
		It only causes sweating	89	21.9%
8	How can hairdressers can reduce their risk of developing hand eczema?	By using gloves and moisturizing regularly	106	26.1%
		By avoiding all chemicals	155	38.2%
		By only washing hands once a day	82	20.2%
		By avoiding hair care altogether	63	15.5%
9	What is the most recommended way to treat hand eczema for hairdressers experiencing symptoms?	Using over-the-counter creams like corticosteroids	123	30.3%
		Ignoring the symptoms	56	13.8%
		Washing hands more frequently	133	32.8%
		Using harsh soaps	44	10.8%
		Hand sanitizers	50	12.3%
10	How can hairdressers recognize the early signs of hand eczema?	Redness, itching, and blisters on the skin	126	31.1%
		A sudden increase in hair loss	29	7.1%
		Excessive sweating	110	27.1%
		Fever and body aches	123	30.3%
		Using alcohol-based hand sanitizers	18	4.4%
11	Which of the following factors should hairdressers avoid to prevent hand eczema?	Exposure to water and chemicals	103	25.4%
		Wearing cotton gloves	81	20%
		Working in hot environments	157	38.6%
		Using hand creams after work	65	16%
	Mean knowledge status (n, %)	Good knowledge	122	30.1%
		Poor knowledge	284	69.9%

#### Attitude of hair dressers.

In this study, 104 (25.6%) of hair dressers had positive attitude towards hand eczema, 79 (19.5%) of hair dressers had neutral attitude towards hand eczema and 223 (54.9%) had negative attitude towards hand eczema. Additionally, 128 (31.5%) of hair dressers strongly disagree with hairdressers are more likely to develop hand eczema compared to other professions, 96 (23.6%) of hair dressers disagree with hairdressers are more likely to develop hand eczema compared to other professions (Table 3).

**Table 3:** Attitude towards hand eczema among hair dressers of Debre Berhan city, January 10-February 20, 2025.

S/N	Questions	Strongly Disagree (1) n (%)	Disagree (2) n (%)	Neutral (3) n (%)	Agree (4) n (%)	Strongly Agree (5) n (%)
1	Hairdressers are more likely to develop hand eczema compared to other professions	128 (31.5%)	96 (23.6%)	78 (19.2%)	37 (9.1%)	67 (16.5%)
2	Regular hand washing and use of chemicals contribute to hand eczema	135 (33.3%)	112 (27.6%)	80 (19.7%)	35 (8.6%)	44 (10.8%)
3	I am aware of the risks of chemicals to skin health	142 (35%)	119 (29.3%)	56 (13.8%)	61 (15%)	28 (6.9%)
4	Hand eczema is preventable in hairdressers	79 (19.5%)	73 (18%)	102 (25%)	88 (21.7%)	64 (15.8%)
5	I feel comfortable seeking medical help if I develop hand eczema	122 (30%)	97 (23.9%)	66 (16.3%)	50 (12.3%)	71 (17.5%)
6	I have noticed skin irritation or eczema symptoms	86 (21.2%)	87 (21.4%)	187 (46%)	28 (6.9%)	18 (4.5%)
7	Hand eczema is a serious concern for hairdressers	28 (6.9%)	38 (9.6%)	71 (17.5%)	102 (25%)	167 (41%)
8	Proper hand care can reduce the risk of eczema	157 (38.7%)	103 (25.4%)	67 (16.5%)	31 (7.7%)	48 (11.7%)
9	I would attend workshops on hand eczema prevention	345 (85%)	61 (15%)	-------	-------	-------
	Positive Attitude	Frequency (Percentage)		104 (25.6%)		
	Neutral Attitude	Frequency (Percentage)		79 (19.5%)		
	Negative Attitude	Frequency (Percentage)		223 (54.9%)		

#### Practice of hair dressers.

Out of 406 hair dressers participated in this study, 110 (27.1%) had good practice towards hand eczema with the remaining 296 (72.9%) had poor practice towards hand eczema. In this study, 115 (28.3%) of hair dressers follow any specific skin care or safety protocols to prevent hand eczema whereas 291 (71.7%) don’t (Table 4).

**Table 4:** Practice towards hand eczema among hair dressers of Debre Berhan city, January 10-February 20, 2025.

S/N	Questions	Category	Frequency (n)	Percentage (%)
1	Do you follow any specific skin care or safety protocols to prevent hand eczema?	Yes	115	28.3%
		No	291	71.7%
2	How often do you use hand creams or moisturizers?	Once a day	62	15.3%
		2-3 times a day	78	19.2%
		Only when I feel dryness	37	9.2%
		Never	229	56.4%
3	How often do you wash your hands during your workday?	Once a day	128	31.5%
		2-3 times a day	197	48.5%
		> 5 times a day	81	20%
4	Do you use any moisturizing creams or lotions to prevent hand eczema?	Yes	177	43.7%
		No	229	56.3%
5	Do you wear gloves while performing hair treatments (e.g., colouring, perming)?	Yes	40	9.9%
		No	366	90.1%
6	Do you take any precautions when handling chemicals (e.g., hair dye, bleach)?	Yes	45	11.1%
		No	361	88.9%
7	Have you ever experienced symptoms such as redness, itching, or swelling on your hands after work?	Yes	137	33.7%
		No	269	66.3%
8	Are there any workplace policies regarding hand care or eczema prevention?	Yes	26	6.4%
		No	380	93.6%
9	Do you seek medical advice if you experience hand eczema symptoms?	Yes	231	56.9%
		No	175	43.1%
10	Do you regularly observe and practice safety protocols related to skin health in your work?	Yes	41	10.1%
		No	365	89.9%
	Good Practice		110	27.1%
	Poor Practice		296	72.9%

**Fig 2:**
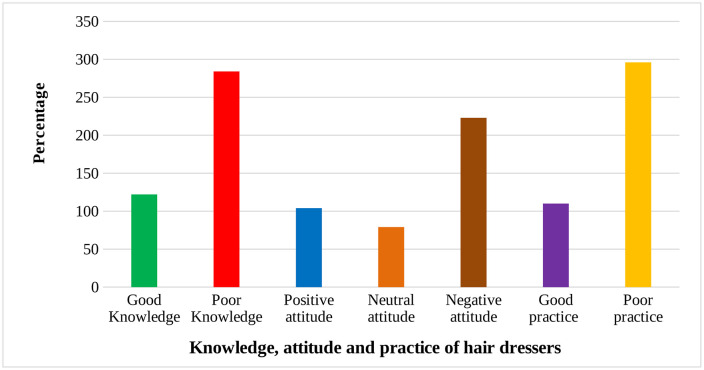
Summary of knowledge, attitude and practice of hair dressers of Debre Berhan city, January 10-February 20, 2025.

In this study, of 406 hair dressers participated in this study, 122 (30.1%) had good knowledge, 104 (25.6%) had positive attitude and 110 (27.1%) had good practice towards hand eczema (Fig 2).

#### Utilization of personal protective equipment and hand washing frequency.

In this study, 269 (66.3%) did not utilize proper personal protective equipment with 253 (62.3%)of hair dressers washed their hands less than or equal to ten times per day (Table 5).

**Table 5:** Behavioural characteristics among hair dressers of Debre Berhan city, January 10-February 20, 2025.

*Variables*	*At least one hand eczema symptom*		*Frequency*	*Percentage*
	Yes	No		
Knowledge				
Good knowledge	62	60	122	30.1%
Poor knowledge	234	50	284	69.9%
Attitude				
Positive attitude	44	50	104	25.6%
Neutral attitude	37	42	79	19.5%
Negative attitude	105	128	223	54.9%
Practice				
Good practice	68	42	110	27.1%
Poor practice	106	190	296	72.9%
Utilization of personal protective equipment				
Yes	51	86	137	33.7%
No	204	65	269	66.3%
Hand washing frequency per day				
≤ 10 times per day	201	52	253	62.3%
> 10 times per day	65	88	153	37.7%

**Fig 3:**
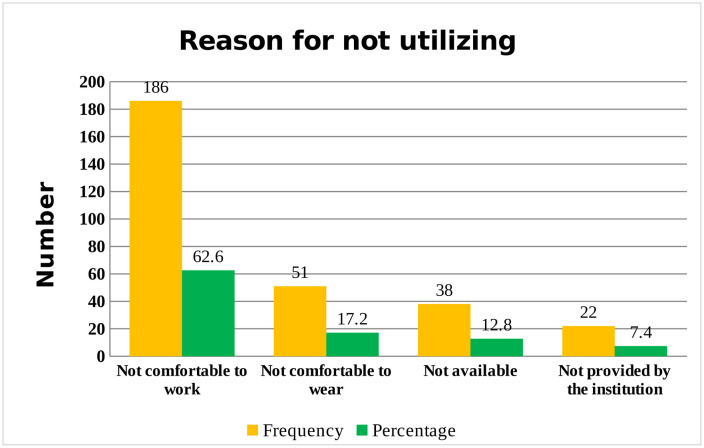
Reason for not utilizing personal protective equipment’s among beauty salon hair dressers of Debre Berhan city, January 10-February 20, 2025.

Regarding the reason for not utilizing personal protective equipments, 186 (62.6%) of hair dressers did not utilize personal protective equipment since it was not comfortable for work (Fig 3).

### Occupational characteristics of hair dressers

Out of 406 hair dressers participated in this study, 250 (61.6%) of hair dressers had worked for less than five years. In addition to this, 210 (51.8%) of hair dressers had worked for less than five days per week (Table 6).

**Table 6:** Occupational related characteristics among hair dressers of Debre Berhan city, January 10-February 20, 2025.

*Variables*	*At least one hand* *eczema symptom*		*Frequency*	*Percentage*
	Yes	No		
Work experience (years)				
≤ 5 years	114	136	250	61.6%
> 5 years	91	65	156	38.4%
Working days per week (days)				
< 5 days	115	95	210	51.8%
5-6 days	26	44	70	17.2%
> 6 days	69	57	126	31%
OHS training				
No	233	76	309	76.1%
Yes	27	70	97	23.9%

OHS = Occupational Health and Safety.

### Prevalence of hand eczema among hair dressers

**Fig 4:**
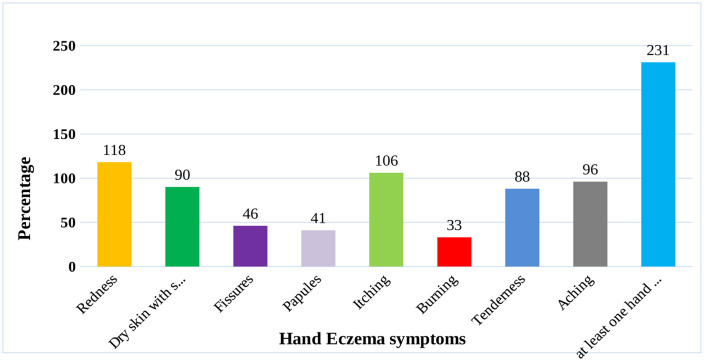
Prevalence of hand eczema among hair dressers of Debre Berhan city, January 10-February 20, 2025.

The prevalence of hand eczema among hair dressers in this study was 56.9% (95% CI: 49.889–61.6) in which 231 (56.9%) hair dressers had at least one hand eczema symptoms. The prevalence of redness and itching among hair dressers was 118 (29.1%) (95% CI: 22.7–34.5) & 106 (26.1%) (95% CI: 21.2–32.3) respectively. Moreover, the prevalence of dry skin with scaling, tenderness and aching among hair dressers was 90 (22.2%) with 95% CI (17.1–26.8), 88 (21.7%) with 95% CI (18.3–25.6%) and 96 (23.6%) with 95% CI (16.8–27.7) respectively (Fig 4).

### Work place observation

An observational assessment revealed that, out of 140 beauty salon institutions included in this study, water was accessible in only 86 (61.4%) of beauty salon institutions. Again out of 140 beauty salon institutions included in this study, majority 110 (78.6%) were containers and 30 (21.4%) were building in their structure. Water was accessible in the house of all beauty salon hair dressing workers. However, soap was not available for hand washing in 105 (75%) of households whereas only 35 (25%) of households had soap for hand washing. Moreover, all of beauty salon hair dressers did not follow proper hand washing technique during practice of hand washing. The overall cleanliness of beauty salon was good in 65 (46.4%) of beauty salon institutions whereas the remaining 75 (53.6%) of beauty salon institutions was poor in their cleanliness.

### Factors associated with hand eczema

The result of multivariable binary logistic regression revealed that not utilizing personal protective equipment, low hand washing frequency per day, and not taking occupational health and safety training were statistically significant predictors for the development of hand eczema symptoms among hair dressers at P value of less than 0.05 (P < 0.05) provided that the effect of other variables were adjusted. Hair dressers who had poor knowledge were 2.89 (AOR = 2.89, 95% CI; 1.199–4.963) times more likely to have at least one hand eczema symptoms as compared to those hair dressers who had good knowledge. Additionally, hair dressers who did not utilize personal protective equipment were 3.8 (AOR = 3.8, 95% CI; 2.183–7.012) times more likely to have at least one hand eczema symptoms as compared to those hair dressers who utilize personal protective equipment provided that the effect of other variables were adjusted. Again, hair dressers who did wash hands less than or equal to 10 times per day were 3.4 (AOR = 3.4, 95% CI; 1.399–6.433) times more likely to have at least one hand eczema symptoms as compared to those hair dressers who wash hands greater than 10 times per day provided that the effect of other variables were adjusted. Finally, hair dressers who did not take occupational health and safety training were 4.8 (AOR = 4.8, 95% CI; 2.617–8.709) times more likely to have at least one hand eczema symptoms as compared to those hair dressers who take occupational health and safety training provided that the effect of other variables were adjusted (Table 7).

**Table 7:** Multivariable binary logistic regression on factors associated with hand eczema among beauty salon hair dressers of Debre Berhan city, January 10-February 20, 2025.

*Variables*	*At least one hand eczema symptom*		*AOR (95% CI)*	*P-value*
	Yes	No		
Educational Status				
Able to read & write	55	32	0.66 (0.176- 1.78)	0.325
Primary education	93	56	0.58 (0.181- 1.44)	0.185
Secondary education	58	48	0.87 (0.307- 2.491)	0.802
Diploma and above	28	36	1	-
Knowledge				
Poor knowledge	234	50	2.89 (1.199-4.963)	0.000*
Good knowledge	62	60	1	-
Utilization of personal protective equipment				
No	51	86	3.8 (2.183- 7.012)	0.000*
Yes	204	65	1	-
Hand washing frequency per day				
≤ 10 times per day	201	52	3.4 (1.399- 6.433)	0.000*
> 10 times per day	65	88	1	-
Work experience (years)				
≤ 5 years	114	136	1	-
> 5 years	91	65	0.83 (0.289- 2.551)	0.622
OHS training				
No	233	76	4.8 (2.617- 8.709)	0.000*
Yes	27	70	1	-

OHS = Occupational Health and Safety training; * = significant level at p<0.05 and AOR = Adjusted Odds Ratio.

## Discussion

It was found that, the overall prevalence of hand eczema among beauty salon hair dressers of Debre Berhan city was 56.9%. The finding of this study was in line with the study that had been done in North East German in which point prevalence of (mostly slight) irritant skin changes of the hands increased from 35.4% in the initial examination to 47.5% in the intermediate examination and to 55.1% in the final examination [20]. Moreover, the present study was in line with the a systematic review and meta-analysis conducted in Croatia in which the overall prevalence of hand eczema among hair dressers was 50.3% [21]. However, the finding of the present study was relatively higher than study conducted in Ethiopia (21.5%) [16], South–South Nigeria (34.3%) [6], Iran (27.8%) [12], Danish (34.5%) [22] and Denmark (38.2%) [11]. The difference might be because of the disparity in study setting, large sample size South-South Nigeria (105) and Iran (385). The disparity might also be during to hand washing practice in which soap was not available for hand washing in 105 (75%) of households and almost all of beauty salon hair dressers did not follow proper hand washing technique during practice of hand washing in the current study. Moreover, the difference might be due to the overall cleanliness of beauty salon in which the overall cleanliness of beauty salon which is good was 65 (46.4%) in the current study. Furthermore, the variation might be due to the difference in atopic history and informal type of beauty salon in which majority of hair dressers might had atopic history and majority of hair dressing beauty salon might be informal.

The finding of this study was relatively lower than study conducted in United Kingdom (72.7%) [10] and another study conducted in United Kingdom (70%) [9]. The variation might be due to the difference in age in which 76.4% of hair dressers were adult in the current study. Again, the difference might be due to the difference in work experience in which majority, 61.6% of hair dressers had work experience of ≤ 5 years since workers with long work experience might exposed more. The inconsistency might be due to the variation in occupational health and safety training in which only 18.8% of hair dressers had taken occupational health and safety training in United Kingdom [10]. But about 23.9% of hair dressers had occupational health and safety training in the present study. The result of the current study showed that hair dressers who had poor knowledge were 2.89 times more likely to have at least one hand eczema symptoms as compared to those hair dressers who had good knowledge. In this study, only 30.1% of hair dressers had good knowledge. The findings was comparable with the study conducted in Egypt [3] in which 33.5% had good knowledge. The reason might be due to shortage of occupational health and safety training in which only 23.9% of hair dressers had received occupational health and safety training in the present study and about 30% received the training in Egypt. However, the result was lower than study conducted in South-South Nigeria [6] where 55.2% of hair dressers had good knowledge towards hand eczema. The difference might be due to the variation in educational status of hair dressers where 91.5% of hair dressers had completed secondary education and above in South-South Nigeria whereas only 41.9% had completed secondary education and above. Hair dressers who did not utilize proper personal protective equipment were 3.8 times more likely to develop at least one hand eczema symptoms than those hair dressers who utilize proper personal protective equipment. The current study revealed that 33.7% of hair dressers utilize proper personal protective equipment. The finding was comparable with the study conducted in India (31.7%) [23], Adryaman Province, Turkey (38.7%) [24] and Denmark (36.4%) [25] of hair dressers utilize proper personal protective equipment. This might be because of personal protective equipment was not-comfortable for work in which 186 (62.6%) of hair dressers in the current study did not utilize personal protective equipment due to non-comfortable nature of personal protective equipment followed by not comfortable to wear 51 (17.2%). Moreover, the comparison might also be due to only 23.9% of hair dressers had taken occupational health and safety training in the present study which leads to poor utilization of personal protective equipment by hair dressers.

In the present study, hand washing frequency per day was significantly associated with hand eczema symptoms. Hair dressers who had hand washing frequency of less than or equal to 10 times per day were 3.4 times more likely to develop at least one hand eczema symptoms than those who washed their hand greater than ten times per day. The result was in agreement with meta-analysis study conducted across different countries in which workers who wash their hands < 8 times per day were 1.46 times more likely to develop confirmed hand eczema as compared to workers who wash their hands 8–10 times per day. The study also reported that workers who wash their hands < 15 times per day were 1.58 times more likely to develop confirmed hand eczema as compared to workers who wash their hands 15–20 times per day [26]. The agreement might be due to the fact that proper hand washing is crucial for hairdressers in reducing cross-contamination, promoting cleanliness, and safeguarding the health of both the hairdresser and their clients. The findings of this study also showed that about 37.7% of hair dressers wash their hands greater than ten times per day. The result was comparable with the findings of the study conducted in Adryaman Province, Turkey in which 24% of hair dressers wash hands only when their hands get dirty [24]. Finally, the findings of the current study revealed that occupational health and safety training was significantly associated with hand eczema symptoms among hair dressers. In this study, hair dressers who had no OHS training were 4.8 times more likely to develop at least one hand eczema symptoms than those hair dressers who had taken OHS training. The current study also revealed that only 23.9% of hair dressers had taken OHS training. The findings of the current study was in agreement with the study conducted in Egypt in which only 30% of hair dressers hand taken OHS training [3]. The findings of the current study was also in agreement with the study conducted in Western Nepal in which only 30% of hair dressers had attended training courses which were both professional and safety directed [27]. The agreement might be due to the fact that OHS training is vital for hairdressers to prevent hand eczema, a common condition caused by prolonged exposure to chemicals, water, and repetitive hand movements. Through proper training, hairdressers learn the importance of using protective gloves when handling harsh products like dyes, shampoos, or chemicals, which can irritate the skin and lead to eczema.

## Limitations of the study

This study has some limitations. Respondents recall bias for questionnaire was minimized by a technique (i.e., decomposition of the question in to small and concrete option). But hair dressers still had recall bias since the study was based on self-reported data. Due to cross-sectional study design, cause and effect relationship was not assessed. Due to the impact of pathogenic microorganisms, hair dressers with confirmed hand eczema symptoms may stop the profession before this study.

## Conclusions

The overall prevalence of hand eczema among beauty salon hair dressers of Debre Berhan city was high. Poor knowledge, not utilizing proper personal protective equipment while at work, low hand washing frequency per day and not taking occupational health and safety training were associated factors with the development of hand eczema symptoms. Hair dressers should utilize proper type of Personal Protective Equipment (PPE) before starting any activities in the work place. Owners should improve the quality of working environment with assured quality, provide proper PPE to reduce the prevalence of hand eczema among hair dressers. More importantly, inclusion of hand eczema education in Technical and Vocational Educational and Training (TVET) or policy-level interventions would enhance occupational health awareness, early prevention strategies and long-term skin protection practices among hairdressers. Furthermore, Researchers should conduct longitudinal study to assess the cause-effect relationship between pathogenic microorganisms in beauty salon and hand eczema symptoms.

## Supporting information

S1 Data Data set. (XLSX)

S1 File Supplementary information. (DOCX)

## Abbreviations

AOR: Adjusted Odds Ratio
HE: Hand Eczema
MSMEs: Micro Small and Medium Enterprises
OHS: Occupational Health and Safety
PPE: Personal Protective Equipment
SDG: Sustainable Development Goals
SE: Standard Error
VIF: Variance Inflation factor.
