# Supplementary material for: Self-reported prevalence of hand eczema and associated factors among hair dressers of Debre Berhan City in North Eastern Ethiopia
Source: PLoS One. 2025 Nov 25;20(11):e0336974. doi: 10.1371/journal.pone.0336974 (PMC12646402; doi:10.1371/journal.pone.0336974)
Supplement: S1 File — (DOCX) [file pone.0336974.s002.docx]

# **Annexes**

## Information Sheet for Interview of Participants (English version)

**Greetings!!**

My name is __________________. I am working as a data collector for the study conducted in this study by **Belachew Tekleyohannes** who is lecturer and researcher working at department of Environmental Health Science. I kindly request you to give me your attention to explain you about the question that I ask you.

**The title of this study**: Self-reported prevalence of hand eczema and associated factors among beauty salon hair dressers of Debre Berhan city, January 10-February 20, 2025

**Purpose of the study:** Self-reported prevalence of hand eczema and associated factors among beauty salon hair dressers of Debre Berhan city, January 10-February 20, 2025

**Procedure of the study with duration:** I will be assessing hand eczema symptoms by using questionnaires that needs your full cooperation and this may take about 20 to 30 minutes.

**Benefit and risk of the study:** There is no risk of participating in this study, since the study does not need collecting any samples that create risks on participants. There would have no any direct benefits and treatment for being study participant but indirectly the findings from this research will important for improving occupational health safety practice, information for the similar institutions and for scientific knowledge.

**Confidentiality & right of participants:** All information forwarded will be kept confidential and names will not be written. Giving permission for this study is voluntary. You have the right to permit or not for this study. If you decide to permit the study, you have the right to terminate the study at any time if you consider something related to the study is wrong. If you have any question about the research you may contact **Belachew Tekleyohannes** (Principal Investigator) at Debre Berhan Health Science College, Department of Environmental Health (phone number: +251-967-51-07-54).

## Informed Consent (English version)

Detail information about the study was explained to me. I have understood that the main objective of this study is to assess self-reported prevalence of hand eczema, and associated factors among beauty salon hair dressers of Debre Berhan city, January 10-February 20, 2025. In addition, I understand about how the data collection is proceeding and the time it takes to complete the data collection. I also understand that the research imposes no risk on me. I assured that there would be confidentiality of my response, photo graph and collected data used only for the study. It also explained to me that I have the right to stop participation at any time. In addition, I understood that participating in this study is important for scientific knowledge and base for further study. Therefore, I have now consented to participate in the study by signing this form.

**Signature of participants**__________________Date: __________________

**Name and signature of data collectors**__________________________ Date: ___________

## Questionnaire (English version)

Debre Berhan Health Science College, Department of Environmental Health: A questionnaire designed to estimate self-reported prevalence of hand eczema and its associated factors among beauty salon hair dressers in Debre Berhan city, North East Ethiopia, 2025.

1. Date of data collection __________________
2. Questionnaire Code _________________
3. Name of sub city __________________
4. Interviewer Name __________________
5. Supervisors Name _________________
6. Rank of the interview: Completed Partially completed

| **Section 1: Demographic characteristics** | | | | | | | |
| --- | --- | --- | --- | --- | --- | --- | --- |
| 1. What is your age? | | | |  | | | |
| 1. What is your sex? | | | | 1. Male 2. Female | | | |
| 3. What is the highest level of education you have completed? (Choose one only) | | | | 1. ☐ No School  2. ☐ Primary School  3. ☐ High School  4. ☐ College/Tertiary | | | |
| 4. Type of employment | | | | 1. ☐ Self-employed  2. ☐ Working for someone | | | |
| 5. Where did you learn how to perform your job as a hairdresser? (Choose only one) | | | | 1. ☐ Hairdressing school  2. ☐ Friend/Family  3. ☐ On the job | | | |
| 6. What is your marital status? | | | | 1. Single 2. Married 3. Divorced | | | |
| 7. What is your household size? | | | |  | | | |
| **Section 2: Personal Protective Equipment’s and Hand Washing Frequency** | | | | | | | |
| 1 | Is there any personal protective equipment in your institution? | | | | 1.☐ Yes 2. ☐ No | |  |
| 2 | If yes, do you usually wear personal protective devices while at work? | | | | 1.☐ Yes 2. ☐ No | |  |
| 3 | What type of protective devices did you use? (more than one response is possible) | | | | . . . | |  |
| 4 | If no, select the most appropriate reasons for not using PPE? | | | | 1.☐ Yes 2. ☐ No | |  |
|  | 1. Not available | | | | 1.☐ Yes 2. ☐ No | |  |
|  | 1. Not comfortable for work | | | | 1.☐ Yes 2. ☐ No | |  |
|  | 1. Not comfortable to wear | | | | 1.☐ Yes 2. ☐ No | |  |
|  | 1. Not provided by institution | | | | 1.☐ Yes 2. ☐ No | |  |
|  | 1. The dust is not harmful | | | | 1.☐ Yes 2. ☐ No | |  |
|  | 1. Others (specify) . | | | |  | |  |
| 5 | Do you wash your hands? | | | | 1.☐ Yes 2. ☐ No | |  |
| 6 | If yes, How many times do you wash your hands per day? | | | | times per day | |  |
| **Section 3: Occupational factors** | | | | | | | |
| 1 | For how long have you been working in this beauty salon? | | | | Years | |  |
| 2 | For how many working days per week you have been working in this factory? | | | | days/week | |  |
| 3 | Do you take occupational health and safety training after you start this profession? | | | | 1.☐ Yes 2. ☐ No | |  |
| 4 | If yes, do you have certificate? | | | | 1.☐ Yes 2. ☐ No | |  |
| **Section 4: Nordic occupational skin questionnaire will be used for measurement of hand eczema and hairdressing guidelines 2015** | | | | | | | |
| 1. Have you ever had an itchy rash that has been coming and going for at least 1 or 3 months, and at some time has affected skin creases? | | | 1.☐ Yes | | | | 2. ☐ No |
| 2. Have you noticed that contact with certain materials, chemicals or anything else in your work makes your eczema worse? | | | 1.☐ Yes | | | | 2. ☐ No |
| 1. Has eczema on your hands affected your life and daily activities in your occupation in any way? | | | 1.☐ Yes | | | | 2. ☐ No |
| 1. Have you visited a doctor because of the itchy wheals (urticaria) on your hand? | | | 1.☐ Yes | | | | 2. ☐ No |
| 5. Has your eczema had a negative influence on your financial situation (medical and other linked expenses, lost workdays, work capacity? | | | 1.☐ Yes | | | | 2. ☐ No |
| 6. When did you last have these itchy wheals on your hands? | | | ☐ During the past 1 month  ☐ 1 month to 3 months ago  ☐ Over a year ago | | | |  |
| 7. Have you had any of the following symptoms on your hands in the past 1 or 3 months and years ago? | | | ☐ no symptoms during the past 12 months  ☐ redness  ☐ dry skin with scaling/flaking  ☐ fissures or cracks  ☐ weeping or crusts  ☐ tiny water blisters (vesicles)  ☐ papules  ☐ rapidly appearing itchy wheals/welts  ☐ itching  ☐ burning, prickling, or stinging  ☐tenderness  ☐aching or pain | | | |  |
|  | | **Section 5: Observational checklist questions** | | | |  | |
| 1 | | Workers get water accessibility near to the working house? | | | | Yes No | |
| 2 | | Type of hair dressing house (Choose one only) | | | | 1. ☐ Building  2. ☐ Cargo container  3. ☐ Shack  4. ☐ In the street | |
| 3 | | Is there water accessibility in the house? | | | | Yes No | |
| 4 | | Is there soap near to the water? | | | | Yes No | |
| 5 | | Workers apply proper procedure techniques? | | | | Yes No | |
| 6 | | Is the working station and tools are clean? | | | | Yes No | |

**Knowledge of hair dressers**

| **S/N** | **Questions** | **Category** |
| --- | --- | --- |
| 1 | What is hand eczema? | Causing the skin on the hands to be dry and cracked |
|  |  | Skin irritation causing redness, itching, and peeling |
|  |  | A fungal infection affecting the hands |
|  |  | A viral rash on the hands |
| 2 | Which of the following is a common cause or trigger for hand eczema? | Exposure to chemicals used in hairdressing |
|  |  | Lack of hand-washing |
|  |  | Exposure to heat only |
|  |  | Infection by bacteria |
| 3 | In which of the following professions is hand eczema most common? | Office workers |
|  |  | Hairdressers and cleaners due to frequent exposure to water, soaps, and chemicals |
|  |  | Teachers |
|  |  | Drivers |
| 4 | What are common symptoms of hand eczema? | Itching, redness, dryness, and sometimes blisters or cracks |
|  |  | Pain and swelling only |
|  |  | Fever and rash |
|  |  | No symptoms, only pain |
| 5 | Which of the following environmental factors increases the risk of hand eczema among hairdressers? | Dry and cold climate |
|  |  | Exposure to humidity and harsh chemicals in hair dyes and treatments |
|  |  | High altitude |
|  |  | Only exposure to sunlight |
| 6 | Which of the following is a significant risk factor for hand eczema in hairdressers? | Wearing gloves for protection |
|  |  | Prolonged exposure to water and irritants like hair dyes |
|  |  | Working in air-conditioned environments |
|  |  | Not using hair products |
| 7 | How does constant exposure to water and chemicals affect a hairdresser’s skin? | It can cause skin dryness and irritation, which may lead to hand eczema |
|  |  | It has no impact on the skin |
|  |  | It helps keep the skin moisturized |
|  |  | It only causes sweating |
| 8 | How can hairdressers can reduce their risk of developing hand eczema? | By using gloves and moisturizing regularly |
|  |  | By avoiding all chemicals |
|  |  | By only washing hands once a day |
|  |  | By avoiding hair care altogether |
| 9 | What is the most recommended way to treat hand eczema for hairdressers experiencing symptoms? | Using over-the-counter creams like corticosteroids |
|  |  | Ignoring the symptoms |
|  |  | Washing hands more frequently |
|  |  | Using harsh soaps |
|  |  | Hand sanitizers |
| 10 | How can hairdressers recognize the early signs of hand eczema? | Redness, itching, and blisters on the skin |
|  |  | A sudden increase in hair loss |
|  |  | Excessive sweating |
|  |  | Fever and body aches |
|  |  | Using alcohol-based hand sanitizers |
| 11 | Which of the following factors should hairdressers avoid to prevent hand eczema? | Exposure to water and chemicals |
|  |  | Wearing cotton gloves |
|  |  | Working in hot environments |
|  |  | Using hand creams after work |

**Attitude of hair dressers**

| **S/N** | **Questions** | **Strongly Disagree (1)**  **n (%)** | **Disagree (2)**  **n (%)** | **Neutral (3)**  **n (%)** | **Agree (4)**  **n (%)** | **Strongly Agree (5) n (%)** |
| --- | --- | --- | --- | --- | --- | --- |
| 1 | Hairdressers are more likely to develop hand eczema compared to other professions |  |  |  |  |  |
| 2 | Regular hand washing and use of chemicals contribute to hand eczema |  |  |  |  |  |
| 3 | I am aware of the risks of chemicals to skin health |  |  |  |  |  |
| 4 | Hand eczema is preventable in hairdressers |  |  |  |  |  |
| 5 | I feel comfortable seeking medical help if I develop hand eczema |  |  |  |  |  |
| 6 | I have noticed skin irritation or eczema symptoms |  |  |  |  |  |
| 7 | Hand eczema is a serious concern for hairdressers |  |  |  |  |  |
| 8 | Proper hand care can reduce the risk of eczema |  |  |  |  |  |
| 9 | I would attend workshops on hand eczema prevention |  |  |  |  |  |

**Practice of hair dressers**

| **S/N** | **Questions** | **Category** |
| --- | --- | --- |
| 1 | Do you follow any specific skin care or safety protocols to prevent hand eczema? | Yes |
|  |  | No |
| 2 | How often do you use hand creams or moisturizers? | Once a day |
|  |  | 2-3 times a day |
|  |  | Only when I feel dryness |
|  |  | Never |
| 3 | How often do you wash your hands during your workday? | Once a day |
|  |  | 2-3 times a day |
|  |  | > 5 times a day |
| 4 | Do you use any moisturizing creams or lotions to prevent hand eczema? | Yes |
|  |  | No |
| 5 | Do you wear gloves while performing hair treatments (e.g., coloring, perming)? | Yes |
|  |  | No |
| 6 | Do you take any precautions when handling chemicals (e.g., hair dye, bleach)? | Yes |
|  |  | No |
| 7 | Have you ever experienced symptoms such as redness, itching, or swelling on your hands after work? | Yes |
|  |  | No |
| 8 | Are there any workplace policies regarding hand care or eczema prevention? | Yes |
|  |  | No |
| 9 | Do you seek medical advice if you experience hand eczema symptoms? | Yes |
|  |  | No |
| 10 | Do you regularly observe and practice safety protocols related to skin health in your work? | Yes |
|  |  | No |
